# The protective effect of *Liza klunzingeri *protein hydrolysate on carbon tetrachloride-induced oxidative stress and toxicity in male rats

**DOI:** 10.22038/ijbms.2019.33201.7927

**Published:** 2019-10

**Authors:** Sana Rabiei, Masoud Rezaei, Zahra Abasian, Mohammad Khezri, Mehdi Nikoo, Mahmoud Rafieian-kopaei, Maryam Anjomshoaa

**Affiliations:** 1 Department of Seafood Processing, Faculty of Marine Sciences, Tarbiat Modares University, Noor, Iran; 2 Medical Plants Research Center, Basic Health Sciences Institute, Shahrekord University of Medical Sciences, Shahrekord, Iran; 3 Department of Pathobiology and Quality Control, Artemia and Aquaculture Research Institute, Urmia University, Urmia, Iran; 4 Department of Anatomical Sciences, Faculty of Medicine, Shahrekord University of Medical Sciences, Shahrekord, Iran

**Keywords:** Antioxidant activity Cytotoxicity, Liza klunzingeri, Oxidative stress, Protein hydrolysate

## Abstract

**Objective(s):**

Today, consumers are looking for food products providing health benefits in addition to meeting the basic nutritional needs of the body. This study aimed to evaluate the antioxidant and cytotoxic effects of *Liza klunzingeri *protein hydrolysate both *in vivo *and *in vitro*.

**Materials and Methods:**

Fish protein hydrolysate (FPH) was prepared using enzymatic hydrolysis with papain. *In vitro* antioxidant activity was assessed using five different antioxidant assays. The cytotoxic effect on 4T1 cell line was evaluated using the MTT assay. The distribution of the molecular weight of FPH was measured using HPLC. In the *in vivo* study, CCl4-exposed Wistar rats were orally treated with FPH (150, 300, and 600 mg/kg) or gallic acid (50 mg/kg) for 28 consecutive days.

**Results:**

Enzymatic hydrolysis gave hydrolysate rich in low molecular weight peptides (<1000 Da) with strong free radicals (ABTS, DPPH, and OH) scavenging activity and cytotoxicity. Treatment of CCl4-exposed rats with all doses of FPH significantly lowered serum aspartate aminotransferase (AST) and alanine aminotransferase (ALT). FPH at doses of 300 and 600 mg/kg significantly decreased lipid peroxidation and improved total antioxidant capacity in serum, liver, and kidney of the CCl4 exposed rats. All doses of L.klunzingeri protein hydrolysate reduced CCl4-induced nitric oxide production of the kidney. Liver histopathological damage caused by CCl4 also ameliorated with all doses of FPH.

**Conclusion:**

*L. klunzingeri *protein hydrolysate can be considered as a functional food to alleviate oxidative stress

## Introduction

Oxidative stress is known as a physiological state in which free radicals and reactive oxygen species (ROS) are overproduced, resulting in damage to various tissue components (1). It is proved that oxidative stress contributes to the development and pathogenesis of different diseases, including diabetes, cancer, atherosclerosis, and age-related neurodegenerative diseases such as Alzheimer’s and Parkinson’s (2). ROS and free radicals are typically generated during cellular metabolism and play a crucial role as signaling messengers in the cellular processes (e.g., growth and proliferation), but excessive production of these reactive components under specific conditions, can induce DNA damage, genomic instability, and altered signaling pathways that contribute to cancer initiation and progression (3).

ROS can be produced from both exogenous origins (e.g., pollutants, radiation, ultraviolet rays, and toxic chemicals) and endogenous origins (e.g., mitochondria, phagocyte, and peroxisome) (4). Different chemicals cause oxidative stress, one of which is carbon tetrachloride (CCl_4_). The damage of the cell’s vital molecules by free radicals derived from reductive dehalogenation of CCl_4_ by cytochrome P450 is the primary mechanism involved in CCl_4_-induced oxidative stress. Evidence suggests that exposure to CCl_4_ induces damage and oxidative stress to the liver and also some other tissues such as the brain, skin, kidneys, and blood (5-7). 

Recent studies show that an antioxidant-rich diet can help strengthen body’s antioxidant defenses to fight oxidative stress and reduce the risk of associated diseases (8, 9). Today, bioactive peptides have drawn increasing attention due to their low molecular weight, easy absorption, significant antioxidant activity, low sensitivity, and high stability under various conditions (10, 11). Bioactive peptides are specific sequences of (10-20) amino acids and remain inactive in the precursor protein sequence until released by proteolytic enzymes (10). Once released, they exhibit numerous physiological functions such as antioxidant (12), antimicrobial (13), anticancer (14), immunomodulatory (15), hypolipidemic (16), and hypoglycemic effects (17). 

Each year, a large proportion of marine organisms, including fish, shellfish, and crustaceans are wasted due to low quality, low consumer preference, and small size (18). According to FAO (Food and Agriculture Organization of the United Nations), the total catch of species of Mugilidae family in the southern coastal waters of Iran was almost 9300 tons in 2016, and *Liza klunzingeri* accounted for 2950 tons of it (19). *L. klunzingeri* is a lowly consumed fish and is generally sold at a low price, which is due to its small size and the presence of distinctive dark peritoneum (20). The use of *L. klunzingeri* for the production of bioactive compounds provides added value and permits the optimal use of marine resources that are decreasing today. The aim of this study was 1) to evaluate the antioxidant and cytotoxic effects *L. klunzingeri* protein hydrolysate *in vitro* and 2) to assess the protective effect of *L. klunzingeri* protein hydrolysate on CCl_4_-induced toxicity and oxidative stress in rats.

## Materials and Methods

***L. klunzingeri*** **preparation and approximate analysis**

Fresh *L. klunzingeri* was purchased from a fish store and transported to the lab on crushed ice. Fish were then washed, filleted, minced, and stored at -20 ^°^C until use. Proximate composition of *L. klunzingeri* mince was determined based on AOAC (Association of Official Analytical Chemists) method (21).

***Enzymatic hydrolysis***

*L. klunzingeri* mince (500 g) was mixed with 100 ml of phosphate buffer (pH= 6) and placed in a water bath at 85 °C for 20 min to deactivate endogenous enzymes. After cooling, the cooked mince was hydrolyzed using papain (enzyme to protein ratio of 1:50) for 180 min at 55 ^°^C. The pH of the reaction mixture was kept at six during the reaction. After 3 hr of hydrolysis, the solution was heated at 95 ^°^C for 15 min to stop the enzymatic reaction. The resulting solution was then centrifuged (8000 g, 30 min, 4 ^°^C) and the supernatant was collected and lyophilized. 

**Figure 1:**
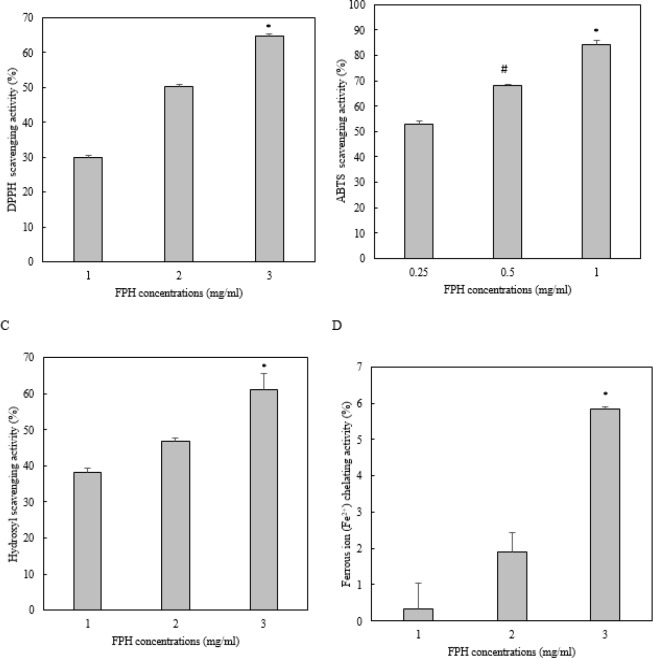
The antioxidant activity of *Liza klunzingeri* protein hydrolysate for DPPH (A), ABTS (B), and hydroxyl (C) scavenging activities and Fe2+ chelating capacity (D). FPH, Fish protein hydrolysate. Data are given as means±SD. In Figures 1-A, C, and D, *shows significant difference (*P*<0.05) between the antioxidant activity of FPH at 3 mg/ml versus that at 1 and 2 mg/ml; In Figure 1-B, * shows significant difference (*P*<0.05) between the antioxidant activity of FPH at 1 mg/ml versus those at 0.25 and 0.5 mg/ml and # shows significant difference (*P*<0.05) between the antioxidant activity of FPH at 0.5 mg/ml versus that at 0.5 mg/ml

**Figure 2:**
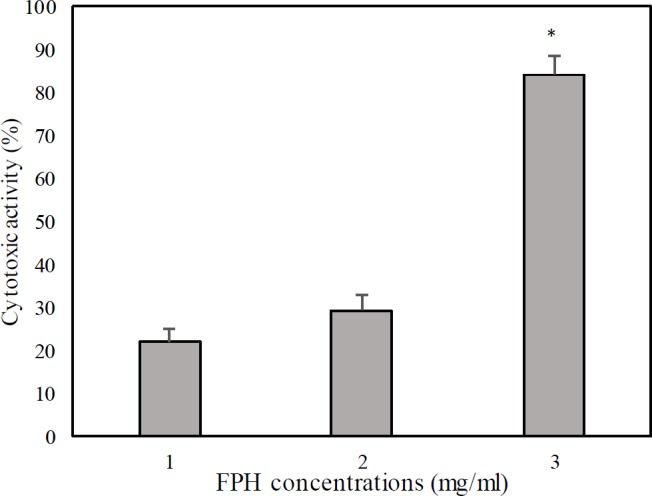
The cytotoxic effect of *Liza klunzingeri* protein hydrolysate on the 4T1 cell line. FPH: Fish protein hydrolysate. Data are given as means±SD. *indicating significant difference (*P*<0.05) between the antioxidant activity of FPH at 3 mg/ml versus those at 1 and 2 mg/ml

**Figure 3:**
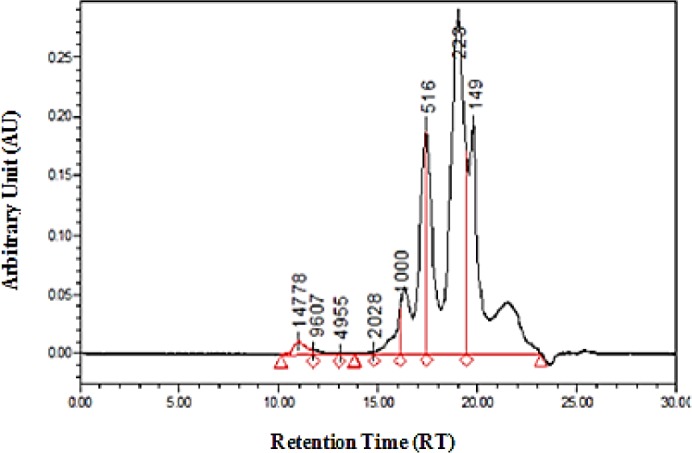
Molecular weight distribution of *Liza klunzingeri *protein hydrolysate

**Figure 4:**
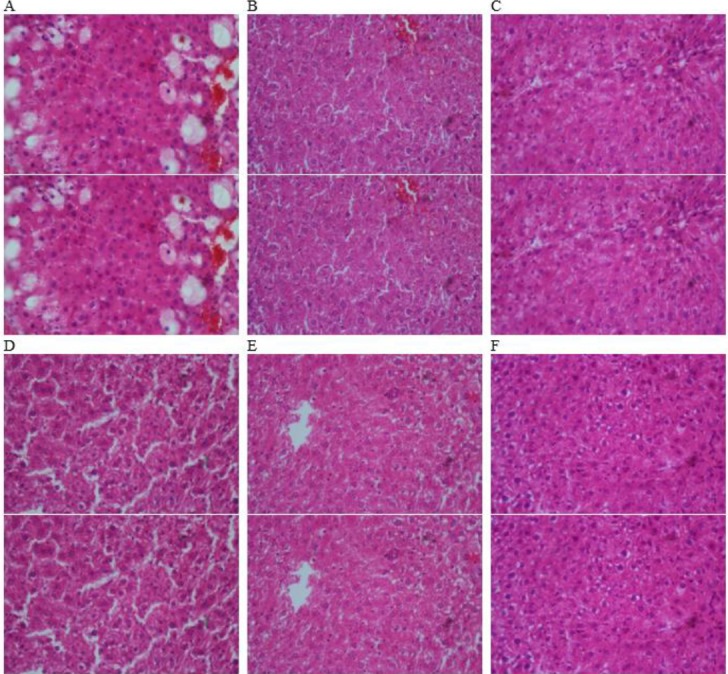
Histopathological features in the liver tissues of the experimental groups. A: Carbon tetrachloride, B: Control, C: Carbon tetrachloride + gallic acid, D: Carbon tetrachloride + FPH (150 mg/kg), E: Carbon tetrachloride + FPH (300 mg/kg), and F: Carbon tetrachloride + FPH (600 mg/kg). (H&E, 100X)

**Figure 5:**
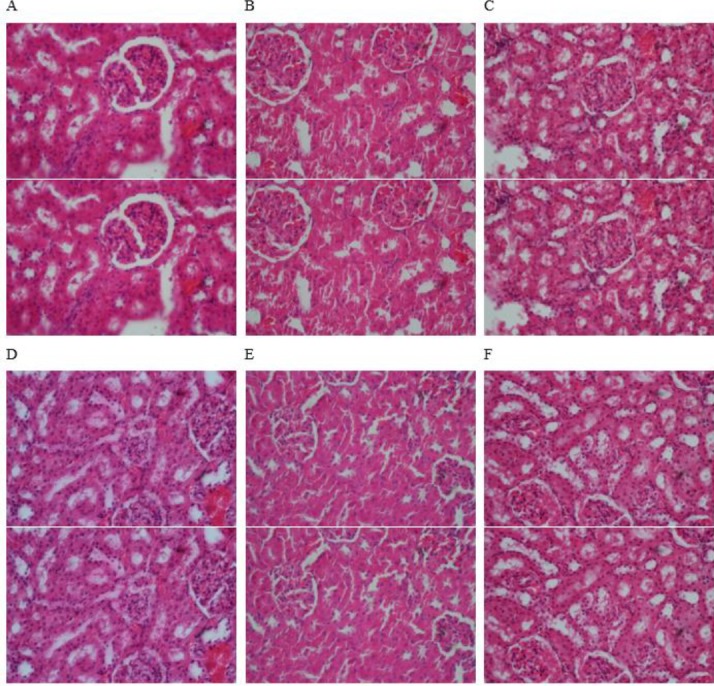
Histopathological features in the kidney tissues of the experimental group. A: Carbon tetrachloride, B: Control, C: Carbon tetrachloride+gallic acid, D: Carbon tetrachloride+FPH (150 mg/kg), E: Carbon tetrachloride+FPH (300 mg/kg) and F: Carbon tetrachloride+FPH (600 mg/kg). (H&E, 100X)

**Table 1:** Proximate composition of unhydrolyzed L. klunzingeri mince and L. klunzingeri protein hydrolysate

Fat (%)	Ash (%)	Protein (%)	Moisture (%)	Sample
0.77±0.46	9.52±0.83	87.84±2.85	1.87±1.72	FPH
2.21±0.53	2.00±0.42	22.46±3.41	73.36±3.90	UFM

Proximate composition was calculated based on the wet matter. Data are given as means ± SD from triplicate determinations (n=3). FPH: Fish protein hydrolysate, UFM: Unhydrolyzed Fish Mince

***DPPH (1,1-diphenyl-2-picrylhydrazyl) radical scavenging activity***

*L. klunzingeri* protein hydrolysate was dissolved in distilled water to obtain the desired concentrations. Then, protein hydrolysate solutions (1 ml) were mixed with 1 ml of 0.1 mM ethanolic solution of DPPH. The absorbance of the mixtures was read at 517 nm after a 15-min incubation at room temperature in the dark. Distilled water was used instead of the protein hydrolysate solution in the preparation of the control sample. Percent scavenging of DPPH radicals was determined using the formula: %DPPH radical scavenging activity= [(A_control_ – A_sample_)/A_control_] × 100. The IC_50_ value was obtained by drawing a graph of the protein hydrolysate concentration (X-axis) against the scavenging activity (Y-axis) (22).

**Table 2:** Amino acid composition of *L. klunzingeri* protein hydrolysate

Amino acid	Gram per 100 g protein
Aspartic acid	2.93908
Glutamic acid	4.91412
Serine	9.50344
Histidine	5.81917
Glycine	1.45979
Threonine	1.08553
Arginine	1.94127
Alanine	1.81932
Tyrosine	8.34432
Cysteine	7.01992
Methionine	1.87524
Valine	6.16082
Phenylalanine	1.11977
Isoleucine	1.45958
Leucine	2.25984
Lysine	2.64536
Proline	2.64536

**Table 3:** Comparison of serum biochemical factors among the study groups

Experimental groups	Blood biochemical factors					
	ALP (IU/I)	ALT (IU/I)	AST (IU/I)	Creatinine (mg/dl)	Urea (mg/dl)	Albumin (mg/dl)
Control	123 ± 291.64	197.50 ±151.11	198.50 ± 29.25	0.65 ± 0.05	37.00 ± 2.85	4.61 ± 0.46
CCl_4_	111 ± 339.82	1456.65 ± 1385.59^*^	507.28 ±281.69^*^	0.65 ± 0.09	40.28 ± 5.62	4.61 ± 0.31
CCl_4_+FPH (150 mg/kg)	107.5 ± 300.52	95.66 ±34.50^#^	148.33 ± 70.58^#^	0.68 ± 0.11	33.66 ± 2.42	4.60 ± 0.18
CCl_4_+FPH (300 mg/kg)	123.57 ± 471.36	83.00 ± 34.67^#^	129.71 ± 46.38^#^	0.67 ± 0.11	36.42 ± 2.76	4.74 ± 0.50
CCl_4_+FPH (600 mg/kg)	119.57 ± 189.26	92.00 ± 38.89^#^	121.28 ± 52.19^#^	0.61 ± 0.03	33.85 ± 5.44	4.45 ± 0.22
CCl_4_+GA (50 mg/KG)	111.85 ± 443.35	99.57 ± 32.54^#^	152.00 ± 20.03^#^	0.58 ± 0.06	31.42 ± 7.63^#^	4.64 ± 0.25

Data are given as means ± SD. * indicating significant difference compared to the values of the control group at *P*<0.05; # indicating significant difference compared to the values of CCl_4_ exposed group at *P*<0.05. FPH: Fish protein hydrolysate, GA: Gallic acid, AST: Aspartate aminotransferase, ALP: Alkaline phosphatase, ALT: Alanine aminotransferase.

**Table 4:** Comparison of Total antioxidant capacity (TAC), Malondialdehyde (MDA) and Nitric oxide (NO•) levels of serum, liver, and kidney among the study groups

Total antioxidant capacity (TAC)							Experimental group	
Serum µmol/g		Kidney µmol/g	Liver µmol/g					
247.42 ± 35.50		314.71 ± 35.10	172.57 ± 36.02				Control	
77.57 ± 24.37^*^		148.57 ± 29.77^*^	63.85 ± 34.41^*^				CCl_4_	
128.85 ± 18.99^#^		165.57 ± 52.98	80.00 ± 40.32				CCl_4_+FPH (150 mg/kg)	
153.28 ± 29.68b^#^		200.57 ± 20.19^#^	116.14 ± 18.91^#^				CCl_4_+FPH (300 mg/kg)	
174.42 ± 56.01^#^		221.57 ± 22.53^#^	127.42 ± 39.19^#^				CCl_4_+FPH (600 mg/kg)	
223.57 ± 24.89^#^		267.14 ± 30.14^#^	130.00 ± 18.79^#^				CCl_4_+GA (50 mg/kg)	
Malondialdehyde (MDA)							
Serum µmol/g	Kidney µmol/g			Liver µmol/g			
250.14 ± 22.86	170.57 ± 19.09	167.42 ± 20.75				Control	
463.00 ± 159.33*	357.42 ± 36.32^*^	355.2 8± 16.22^*^				CCl_4_	
386.42 ± 22.20	266.00 ± 32.39^#^	281.57 ± 33.41^#^				CCl_4_+FPH (150 mg/kg)	
319.85 ± 20.38^#^	259.00 ± 28.00^#^	243.85 ± 49.74^#^				CCl_4_+FPH (300 mg/kg)	
311.71 ± 46.35^#^	206.71 ± 17.89^#^	228.28 ± 20.77^#^				CCl_4_+FPH (600 mg/kg)	
272.28 ± 28.33^#^	189.57 ± 15.26^#^	194.28 ± 40.83^#^				CCl_4_+GA (50 mg/kg)	
Nitric Oxide (NO)							
Serum µmol/g	Kidney µmol/g		Liver µmol/g				
169.33 ± 45.65	117.33 ± 10.70	92.33 ± 14.69				Control	
374.00 ± 69.83^*^	226.50 ± 44.16^*^	254.16 ± 52.09^*^				CCl_4_	
355.50 ± 36.31	173.66 ± 30.25^#^	176.16 ± 18.44				CCl_4_+FPH (150 mg/kg)	
322.16 ± 25.58	100.16 ± 27.12^#^	141.00 ± 37.29				CCl_4_+FPH (300 mg/kg)	
318.50 ± 46.83	140.50 ± 29.22^#^	158.83 ± 147.37				CCl_4_+FPH (600 mg/kg)	
278.66 ± 70.93^#^	137.00 ± 17.41^#^	142.50 ± 32.53				CCl_4_+GA (50 mg/kg)	

Data are given as means ± SD. * indicating significant difference compared to the values of the control group at *P*<0.05; # indicating significant difference compared to the values of CCl_4_ exposed group at *P*<0.05. FPH: Fish protein hydrolysate and GA: Gallic acid.

***Fe*** ^2+^ ***chelating activity***

Briefly, 1 ml of various concentrations of protein hydrolysate and 3.7 ml of distilled water were added to test tubes and mixed with 0.1 ml of 2 mM FeCl_2_ and 0.2 ml of 5 mM ferrozine. After approximately 20 min, the absorbance of the reaction solutions was measured at 562 nm. The control sample was prepared using distilled water in place of the sample. Fe^2+^ chelating activity was obtained using the formula: Fe^2+^ chelating activity (%) = [(A_control _- A_sample_)/A_control_] ×100. The IC_50_ value was obtained by drawing a plot of the chelating activity against the protein hydrolysate concentrations (22).

***ABTS (2,2’-azino-bis(3-ethylbenzothiazoline-6-sulphonic acid)) radical scavenging activity***

In order to prepare ABTS working solution, 5 ml of 7.4 mM ABTS solution was mixed with 5 ml of 2.6 mM potassium persulfate solution and kept at room temperature in the dark for 12 hr. Then, freshly prepared ABTS solution was diluted with methanol to achieve an absorbance of 1.1 ± 0.02 at 734 nm, and 2850 µl of the diluted solution was mixed with 150 µl of protein hydrolysate at various concentrations. After 2 hr incubation at room temperature, the optical absorbance was recorded at 734 nm. The control sample was prepared using 150 µl of distilled water instead of the protein hydrolysate solution. ABTS scavenging activity was determined using the formula: ABTS scavenging activity (%) = [(A_control _- A_sample_)/A_contro_] × 100. The IC_50 _value was obtained by drawing a plot of the scavenging activity against the protein hydrolysate concentrations (22).

***Hydroxyl radical scavenging activity***

First, two milliliters of protein hydrolysate solutions at various concentrations was added to 1 ml of 1.865 mM 1, 10-phenanthroline solution, and mixed well. Then, 1 ml of the resulting solution was mixed with 1 ml of 1.865 mM FeSO_4_ solution and 1 ml of 3% H_2_O_2_ solution. Following the incubation at 37 °C in a water bath for 60 min, the absorbance of the solution was recorded at 536 nm. A blank sample was prepared using the same manner without H_2_O_2 _addition. Negative control was prepared using distilled water instead of the protein hydrolysate. The percentage of hydroxyl radical scavenging activity was obtained from the formula: Hydroxyl radical scavenging activity (%) = [(A_s_-A_n_)/( A_b_-A_n_)] × 100; where A_s_ is the absorbance of the sample, A_n_ is the absorbance of the negative control, and A_b_ is the absorbance of the blank. The IC_50 _value was determined by drawing a plot of hydroxyl radical scavenging activity against the protein hydrolysis concentration (22).

***Evaluation of cytotoxic effects***

The MTT (Thiazolyl blue tetrazolium bromide) assay was applied to evaluate the cytotoxic activity of *L. klunzingeri* protein hydrolysate. 4T_1_ carcinoma cell line was obtained from the National Cell Bank of Iran (Pasteur Institute., Tehran, Iran) and cultured in DMEM (Dulbecco’s Modified Eagle Medium) containing 10% FBS. After reaching around 80% confluence, they were detached by trypsin/EDTA, and the number of 4T_1_ cells was determined using a hemocytometer lam. Then, 200 μl of the suspension containing 15 × 10^3^ cells was added to each well of a 96-well plate. In the next step, the cells were then exposed to 1, 2, and 3 mg/ml of protein hydrolysate for 48 hr. After 48 hr of exposure, the medium was removed and the well washed by PBS. Then 60 μl of MTT solution in PBS (phosphate-buffered saline) was added to each well. The cells were then kept at 37 ^°^C in 5% CO_2_ for 4 hr. After incubation, the medium was removed from the wells, and 150 μl of DMSO was added to each well. The plates were then incubated for 30 min at 37 ^°^C in the dark. Finally, the plates’ absorbance was read at 570 nm using an ELISA reader. The percentage of cytotoxicity was calculated by the formula: Percentage of cell cytotoxicity =× 100]. The IC_50_ value of the sample was determined by plotting the percentage of cytotoxicity against the concentrations.

***Determination of molecular-weight distribution***

The molecular weight distribution of the protein hydrolysate was assessed by gel permeation chromatography using an Agilent 1100 HPLC system (Column: TSK gel 2000 SWXL (300 × 7.8mm), Mobile phase: 40% CH_3_CN (0.5 ml/min), and wavelength 225 nm) (23).

***Determination of the amino acid composition***

Amino acids composition of the hydrolysate was determined using the AOAC method with some modiﬁcations. One hundred and twenty milligrams of lyophilized hydrolysate was digested using 8 ml of 6 M HCl under N_2_ atmosphere at 110 °C for 22 hr. After cooling, the reaction solution was mixed with 4.8 ml of 10 M NaOH and then distilled water was added to obtain a final volume of 25-ml. The mixture was then ﬁltered (Whatman Grade 40) and centrifuged (10,000g, 10 min). Amino acids composition were estimated by using Agilent 1100 HPLC. One microliter of the sample was injected into a Zorbax, 80A C-18 column (column size: 4.0×250mm and particle size: 5μm) at 40 °C with detection at 338 nm. The mobile phase A was 7.35 mM/L of sodium acetate/ triethylamine/ tetrahydrofuran (500:0.12:2.5, v/v/v), adjusted to pH 7.2 using acetic acid, while the mobile phase B (pH 7.2) was 7.35 mM/L of sodium acetate/methanol/ acetonitrile (1:2:2, v/v/v). The amino acids composition of the hydrolysate was expressed as g of amino acids per 100 g of protein (23).

***Evaluation of in vivo antioxidant activity***

*Experimental animals and grouping*

Forty-eight male Wistar rats (aged three months and weighing 200–240 g) were purchased from the Animal Breeding Facility Centre of Pasteur Institute, Karaj, Iran. Animals were kept under standard conditions (21±2 ^°^C and 12:12 hr light/dark cycle) with free access to the same water and food. This study was approved by the Ethics Committee of Shahrekord University of Medical Sciences, and all animal procedures were based on Guidelines for the Care and Use of Laboratory Animals. Rats were randomized into six groups of eight animals. Control group received intraperitoneal (IP) injection of olive oil (1mg/kg) twice a week and normal saline daily by gavage. The model group received an IP injection of CCl_4_ (1 ml/kg, 50% v/v in olive oil) twice a week and 1 ml/kg of normal saline daily by gavage. Intervention groups received CCl_4_ plus daily oral doses of protein hydrolysate (150, 300, and 600 mg/kg). The positive control group received CCl_4_ plus gallic acid at a dose of 50 mg/kg (dissolved in normal saline) daily by gavage. After 28 days of treatment, animals were kept fasting for 12 hr and then sacrificed under general anesthesia by chlorophyll. Blood samples were collected from the hearts of the anesthetized animals. The blood samples were then centrifuged for 15 min at 1500 g, and sera were separated and immediately stored at −70 ^°^C until analysis. Animals were then autopsied, and the liver and kidneys were removed and divided into two sections. One section was fixed in 10% formalin for pathophysiological analysis, and one section was kept at –70 ^°^C for biochemical analysis.

***Measurement of serum biochemical factors***

Aspartate aminotransferase (AST), alkaline phosphatase (ALP), and alanine aminotransferase (ALT) levels, as well as serum urea, creatinine, and albumin, were measured by commercially available kits (Pars Azmoon Co., Tehran, Iran) using Biotecnica autoanalyzer BT-3000.

***Measurement of lipid peroxide levels***

Tissue homogenate/ serum (0.2 ml), acetic acid (1.5 ml of 20%), Thiobarbituric acid (1.5, 0.8%) and Sodium dodecyl sulfate (200 μl, 8.1%) were added to a test tube and mixed well. To the mixture, 700 µl of distilled water was added and heated in a water bath (94–96 ^°^C) for 60 min. Upon cooling with tap water, 1 ml of distilled water and 5 ml of n-butanol/pyridine solution were added to and shaken vigorously. After centrifugation (4000 rpm, 10 min), the optical absorbance of the supernatant was read at 532 nm. Lipid peroxide levels were determined using a standard calibration curve and expressed as a micromole of malondialdehyde (24).

***Measurement of total antioxidant capacity***

Ferric reducing antioxidant power (FRAP) assay was used to measure the total antioxidant capacity of serum and tissue homogenate. The working FRAP reagent was made by mixing 10 ml of 0.25 M acetate buffer (pH= 3.6), 2.5 ml of 10 mM TPTZ (in 40 mM HCl) and 2.5 ml of 20 mM FeCl_3_.6H2O. 1.5 ml of freshly prepared FRAP reagent was mixed with 25 µl of tissue homogenate/serum and incubated at 37 °C for 10 min. Then, the optical absorbance was read at 593 nm (24).

***Measurement of nitric oxide (NO*** ^•^ ***) levels***

Nitric oxide (NO•) is an unstable, very reactive and short-lived free radical which rapidly oxidizes to more stable final metabolites such as nitrates (NO^3-^) and nitrite (NO^2-^); Thus, total NO^2-^ level of serum/tissue homogenate was estimated as an index of NO• production. First, 600 μl of ZnSO_4_ solution (75 mM) was added to 300 μl of serum/tissue homogenate and centrifuged at 1000 g for 5 min at room temperature. The supernatant was removed and incubated with copper cadmium granules in a glycine-NaOH buffer to convert NO^3-^ to NO^2-^. Total NO^2-^ level was determined using the Griess reaction. For this purpose, 1 ml of the sample was mixed with 1 ml of Griess solution (1 ml of 0.5% sulfanilamide and 0.05% n-naphthalene diamine hydrochloride) and kept for 30 min at room temperature in the dark. Then, the absorbance at 545 nm was read (24).

***Histopathological analysis***

Samples of rat liver and kidney were formalin-fixed, processed, and embedded in paraffin. Two-micron sections of samples were then prepared and stained with hematoxylin-eosin (H & E) for histological evaluation under a light microscope.

***Statistical analysis***

SPSS 20 was used to analyze the collected data. Analysis of Variance (ANOVA) followed by Duncan’s test was used to detect statistical differences in means. All data were expressed as mean±SD, and *P*<0.05 was considered statistically significant.

## Results

Figure 1 shows the antioxidant activity of *L. klunzingeri* protein hydrolysate for scavenging of DPPH, ABTS, and hydroxyl free radicals and chelating of Fe^2+^. According to the results, protein hydrolysate obtained by enzymatic hydrolysis of *L. kludingeri* muscle exhibited strong scavenging activity on ABTS radicals (IC_50_= 0.12±0.016 mg/ml), good scavenging activities on DPPH (IC_50_= 2.08 ±0.13 mg/ml) and hydroxyl (IC_50_= 2.07±0.31 mg/ml) radicals, and weak chelating activity on Fe^2+^ (IC_50_= 12.60 ±0.02 mg/ml). *L. klunzingeri* protein hydrolysate also showed strong cytotoxicity on 4T_1 _cancer cell (IC_50_= 2.15 ±0.1, Figure 2).

Table 1 shows the results of the proximate composition of *L. klunzingeri* protein hydrolysate and unhydrolyzed *L. klunzingeri* mince. The protein content of protein hydrolysate and unhydrolyzed mince were 87.84±2.85% (wet basis) and 22.46±3.41% (wet basis), respectively.

The analysis of the molecular weight distribution of *L. klunzingeri* protein hydrolysate using HPLC showed that 95% of the peptides in this sample had a molecular weight of less than 1000 Da. 30.56% of peptides in this sample had a molecular weight of less than 180 Da, 47.26% had a molecular weight of 180–500 Da, and 17.46% had a molecular weight of 500–1000 Da (Figure 3).

The most abundant amino acids in *L. klunzingeri* protein hydrolysate were serine (9.593%), tyrosine (8.43%), cysteine (7.197%), valine (6.60%), histidine (5.81%), and glutamine (4.914%) (Table 2).

*L. klunzingeri* protein hydrolysate was then evaluated for its antioxidant and protective effects against CCL_4_ induced toxicity and oxidative stress. CCl_4_ exposed rats were orally treated with *L. klunzingeri* protein hydrolysate at doses of 150, 300, and 600 mg/kg for 28 days. Table 3 shows the comparison of serum levels of biochemical factors between the study groups. Animals treated with CCl_4_ exhibited a significant increase in serum levels of AST and ALT (*P*<0.05) without any significant changes in the levels of ALP, creatinine, urea, and albumin. Oral treatment of CCl_4_ exposed rats with *L. klunzingeri* protein hydrolysate at three doses and gallic acid caused a significant reduction in the serum levels of AST and ALT (*P*<0.05).

Total antioxidant capacity (TAC), Malondialdehyde (MDA), and Nitric Oxide (NO•) levels of serum, liver, and kidney tissues in experimental groups were represented in Table 4. As shown, CCl_4 _exposure caused a significant decrease in TCA level and a significant increase in MDA and NO• levels of both serum and tissue samples (*P*<0.05). The oral administration of gallic acid and all doses of *L. klunzingeri* protein hydrolysate to CCl_4_ exposed rats led to a significant and dose-dependent increase of the serum TCA level (*P*<0.05). *L. klunzingeri *protein hydrolysate at a dose of 150 mg/kg did not cause any significant changes in the liver and kidney TCA but significantly increased their TCA levels at doses of 300 and 600 mg/kg (*P*<0.05). Oral treatment with *L. klunzingeri* protein hydrolysate at doses of 150, 300, and 600 mg/kg significantly reduced MDA levels in the liver and kidneys, and at doses of 300 and 600 mg/kg in the serum (*P*<0.05). *L*. *klunzingeri *protein hydrolysate at all three doses did not cause any significant changes in the serum and liver NO levels, but significantly decreased kidney NO level (*P*<0.05).

Histological studies showed that treatment with CCl_4_ resulted in localized necrosis, fatty changes, balloon-shaped damage, and liver inflammation and treatment with 150, 300, and 600 mg/kg of protein hydrolysate and gallic acid prevented the damages mentioned above (Figure 4). The kidney tissue in all groups was normal, and no damage was observed (Figure 5).

## Discussion

The present study showed the potential protective effects of *L. klunzingeri* protein hydrolysate on CCl_4_ -induced oxidative stress and toxicity. Elevated serum hepatic biomarkers, AST and ALT activities were observed in CCl_4_ treated rats suggesting damage to hepatocytes and impairment of hepatic function. Histological studies also indicated necrosis, inflammation, and hepatic damage in CCl_4_-treated rats. The administration of *L. klunzingeri* protein hydrolysate to CCl_4_-exposed rats significantly ameliorated these changes in the normal levels. We also observed no significant deterioration of renal function and structure in rats treated with CCl_4_. The toxic effects of CCl_4_ on liver tissue and function has been extensively studied (6, 7, 25). However, only a few studies have been done to investigate CCl_4_-induced renal toxicity (5).

CCl_4_, a potent hepatotoxic agent, is known to cause liver damage by induction of oxidative stress and generation of free radicals. Cytochrome P450 enzymes convert this compound in the endoplasmic reticulum of hepatocytes into trichloromethyl radicals. These radicals are highly reactive and rapidly react with molecular oxygen and produce proxy trichloromethyl radicals. The resulting radicals attack vital biomolecules, including lipids, proteins, and nucleic acids, causing damage and death of hepatocytes. When hepatocyte membrane is damaged by free radicals, various enzymes normally present in the cytosol are released into the bloodstream, thereby causing elevated serum level of liver enzymes (25). In the present study, the increased serum levels of liver enzymes, the elevated oxidative stress markers (MDA and NO• production) and also the decreased antioxidant capacities in the serum, liver, and kidney tissues of CCl_4_-exposed rats confirmed this mechanism. 

So far, a few animal studies have shown that peptides derived from enzymatic hydrolysis of natural protein possess protective effects on oxidative stress *in vivo*. Oral administration of protein hydrolysate of *Sardinella aurita* muscle in rats fed high-fat diet significantly improved the antioxidant defense system (26). *Salaria basilisca* protein hydrolysate also reduced the oxidative stress markers and strengthened the antioxidant defense system in alloxan-induced diabetic mice (27). 

The mechanism by which peptides exert their antioxidant effects has not yet been fully understood, although various *in vitro* studies have shown that peptides and protein hydrolysates exert their effects by scavenging free radicals, metal ion chelation and inhibition of enzymatic (lipoxygenase) and non-enzymatic enzymatic oxidation (28). Also, bioactive peptides have been reported to increase the expression of genes associated with enzymatic and non-enzymatic antioxidants (homoxygenase and ferritin) in cell culture media (29). In the present study, peptides of *L. klunzingeri* muscle decreased free radical-induced lipid peroxidation and also NO levels. *L. klunzingeri* protein hydrolysate also increased the antioxidant capacity of serum and tissue. 

It was reported that the efficacy of bioactive peptides to treat and prevent various diseases depends substantially on their bioavailability (30). In order to exhibit therapeutic benefits, bioactive peptides should resist gastrointestinal digestion, pass through the intestinal epithelium, and reach blood, tissues, and cells (31). Limitations such as physical and chemical instability, degradation by digestive enzymes, inability to cross the intestinal epithelium, accumulation, surface absorption, and immunogenicity affect the biological activities of bioactive peptides (32). It is documented that peptides with 2–6 amino acids are better absorbed in the human digestive tract than proteins and peptides with large molecular weight (33). Short-chain peptides exhibit higher resistance to digestive enzymes and have a greater chance of passing through the gastrointestinal tract (32). In the present study, 95% of peptides in *L. klunzingeri* protein hydrolysate had a molecular weight of less than 500 Da, corresponding to di- and tri-peptides. It seems that the presence of high amounts of short-chain antioxidant peptides in hydrolysate is responsible for the antioxidant activity observed *in vivo*. 

## Conclusion

IIt could be argued that hydrolysate obtained from enzymatic hydrolysis of *L. klunzingeri* muscle is not only the source of high-value protein (protein content: 88.19 ± 2.15%) but also exhibits significant antioxidant effect *in vivo* and can protect the tissues and body against oxidative stress. The considerable *in vivo* efficacy of the hydrolysate may probably be related to the presence of low molecular weight peptides with strong potency of gastrointestinal absorption.
